# Five Aspects Meal Model of Eating Experiences in Older People With Dementia: Theory Analysis and Evaluation

**DOI:** 10.1093/geroni/igaf122.1322

**Published:** 2025-12-31

**Authors:** Zih-Ling Wang, Basia Belza

**Affiliations:** University of Washington School of Nursing, Seattle, Washington, United States; University of Washington, Seattle, Washington, United States

## Abstract

Eating experiences significantly impact the quality of life and nutritional status of people living with dementia (PLWD). The concept of eating experiences encompasses both subjective perceptions and functional aspects of mealtimes, offering a broader perspective than traditional approaches focused solely on nutrition or feeding/eating difficulties. This paper presents a theory analysis of the Five Aspects Meal Model (FAMM) as applied to eating experiences in older people with dementia, using Walker and Avant’s systematic approach. The FAMM provides a comprehensive framework examining five interconnected dimensions of meals: room, meeting, product, management control system, and atmosphere. The analysis examines the model’s origins, meaning, logical adequacy, usefulness, generalizability, parsimony, and testability in the context of eating experiences for PLWD. Findings indicate that the FAMM effectively conceptualizes how physical environment (room), social interactions (meeting), food qualities (product), organizational factors (management control system), and multisensory elements (atmosphere) collectively shape eating experiences. The interrelationships between these five aspects offer valuable insights into enhancing mealtime satisfaction and nutritional intake. While the FAMM has been widely used in hospitality and food service research, it has yet to be fully applied within gerontological nursing, particularly dementia care. This model has significant potential for application in long-term care settings, where holistic mealtime approaches are increasingly recognized as essential to person-centered care. This analysis contributes to nursing science by providing a theoretical foundation for developing comprehensive interventions that address multiple dimensions of eating experiences for PLWD.

